# Plant growth-promoting bacteria in metal-contaminated soil: Current perspectives on remediation mechanisms

**DOI:** 10.3389/fmicb.2022.966226

**Published:** 2022-08-11

**Authors:** Yue Wang, Mathiyazhagan Narayanan, Xiaojun Shi, Xinping Chen, Zhenlun Li, Devarajan Natarajan, Ying Ma

**Affiliations:** ^1^College of Resources and Environment, Southwest University, Chongqing, China; ^2^Division of Research and Innovation, Department of Biotechnology, Saveetha School of Engineering, Saveetha Institute of Medical and Technical Science, Chennai, Tamil Nadu, India; ^3^Department of Biotechnology, Periyar University, Salem, Tamil Nadu, India

**Keywords:** plant growth-promoting bacteria, metal bioavailability, metal detoxification, climatic stresses, bioremediation

## Abstract

Heavy metal contamination in soils endangers humans and the biosphere by reducing agricultural yield and negatively impacting ecosystem health. In recent decades, this issue has been addressed and partially remedied through the use of “green technology,” which employs metal-tolerant plants to clean up polluted soils. Furthermore, the global climate change enhances the negative effects of climatic stressors (particularly drought, salinity, and extreme temperatures), thus reducing the growth and metal accumulation capacity of remediating plants. Plant growth-promoting bacteria (PGPB) have been widely introduced into plants to improve agricultural productivity or the efficiency of phytoremediation of metal-contaminated soils *via* various mechanisms, including nitrogen fixation, phosphate solubilization, phytohormone production, and biological control. The use of metal-tolerant plants, as well as PGPB inoculants, should hasten the process of moving this technology from the laboratory to the field. Hence, it is critical to understand how PGPB ameliorate environmental stress and metal toxicity while also inducing plant tolerance, as well as the mechanisms involved in such actions. This review attempts to compile the scientific evidence on this topic, with a special emphasis on the mechanism of PGPB involved in the metal bioremediation process [plant growth promotion and metal detoxification/(im)mobilization/bioaccumulation/transformation/translocation] and deciphering combined stress (metal and climatic stresses) tolerance.

## Introduction

Soil contaminated with heavy metals has become a serious worldwide problem due to geologic and anthropogenic activities, such as mining, fossil fuel combustion, application of agrochemicals, and so on. As heavy metals are non-biodegradable and extremely persistent in the environment, they can easily accumulate in different foods. Metal contamination of various foods, such as crops, meat, fish, milk, and eggs, threatens food safety. Metals contaminate agricultural soils, irrigation water, plants, and animals, resulting in their incorporation into the food chain and posing a significant threat to human health and ecosystems (Abdel-Rahman, 2022). The major sources of heavy metals and their harmful effects are summarized in Table 1. There are currently numerous methods for controlling heavy metal pollution. The advantages and disadvantages of different techniques are summarized in Table 2. Traditional remediation technologies for contaminated soil, such as cleaning, heat treatment, electrochemistry, and amendment application, often have complex processes that easily destroy soil structure and fertility. They are ineffective for treating both low concentration and large-scale heavy metal contamination in soils. Phytoremediation is a potential solution for remediating metal-polluted soils since it is a cost-effective plant-based approach (Ma et al., 2016a). During global climate change scenarios, plants are more severely and frequently subjected to episodes of climatic stress, such as high temperature, drought, and salinity, limiting their growth and performance. Furthermore, the direct (e.g., competition of ions) or indirect (e.g., alteration of soil physicochemical-biological properties) impact of climate change on metal bioavailability in soils may impede plant adaptation, making them more susceptible to stress and thus limiting the widespread application of phytoremediation (Rajkumar et al., 2013). Plant beneficial microorganisms (PBM), particularly plant growth-promoting bacteria (PGPB) create symbiotic relationships with plants, alleviating the toxicity of heavy metals, promoting multimodal tolerance of plants to metals and climatic stresses, and affecting the bioavailability of metals in soils (Ma et al., 2016b). For instance, PGPB can alleviate metal toxicity and alter metal bioavailability in soils through metal biosorption, bioaccumulation, redox reaction, mobilization, precipitation, and transformation (Ma et al., 2016a). They can also provide plants with multiplex tolerance to a variety of climatic stresses (such as drought and high salinity) by producing 1-aminocyclopropane-1-carboxylate deaminase (ACCD), siderophore, and phytohormone, and dissolving insoluble mineral nutrients (such as nitrogen, phosphorus, and potassium). These PGPB strains could also protect plants from phytopathogens by producing antibiotics and inducing induced systemic resistance (Grover et al., 2021). Understanding the interaction between plants and PBM has lots of potential for accelerating metal phytoremediation under various environmental stressors (e.g., salinity, drought, and extreme temperature). There have been very few investigations on plant-microbe associations for bioremediation of metal-polluted soils under climatic stresses.

**Table 1:** The sources and harmful effects of metals.

Metal	Sources	Harmful effects	Reference
Cd	Electroplate, mine, smelt, fuel, battery, and chemical wastewater discharge	Carcinogenic, bone injury, kidney stone, failure, coughing, emphysema, and headache	Sarwar et al., 2017
Pb	Paint, coating, smelt, hardware storage battery, puffed food, hair dye, and fire coal	Renal failure, cardiovascular disease, mental decline, high blood pressure, and anorexia	Guo et al., 2020
Cu	Metal processing, machinery manufacturing, iron and steel production, and copper-zinc mining	Brain and kidney damage, severe anemia, abdominal pain, and diarrhea	Vardhan et al., 2019
Zn	Zinc mining, smelt, and machinery manufacturing	Carcinogenic, ataxia, depression, and gastrointestinal irritation	Li et al., 2019
Hg	Instrument and meter plant, salt electrolysis, precious metal smelting, cosmetics, lighting lamp, and dental material	Depression, fatigue, hair loss, visual and hearing impairment, ulcer, and kidney damage	Kim et al., 2016
As	Mine, smelt, chemical pharmacy, insecticide, chemical fertilizer, and arsenate drug	Anorexia, gastrointestinal disorders, corneal sclerosis, skin darkening cardiovascular, and respiratory disorder	Balali-Mood et al., 2021
Cr	Steel industry, tanneries, sludge, and solid waste	Bronchopneumonia, chronic bronchitis, diarrhea, emphysema, liver diseases, and renal failure	Sarwar et al., 2017
Ni	Kitchen appliances, surgical instruments, steel alloys, and automobile batteries	Dermatitis, hepatotoxic, lungs, dry cough, and shortness of breath	Rajendran et al., 2021

**Table 2:** Advantages and disadvantages of the available remediation techniques for metal-contaminated soils.

Method	Remediation technique	Advantages	Disadvantages	Reference
Physical remediation	Soil washing	Simple technology	High cost, installing solutions, collection wells, or underground drains may be difficult	Rajendran et al., 2021
	Surface covering	Easy to install, low cost, and high security	Limited to a small area, the soil loses its natural environmental function	Liu et al., 2018
	Soil replacement	Fast to implement and high efficiency	High cost, limited to seriously polluted small-scale soil	Rajendran et al., 2021
	Encapsulation	High security and fast install	High cost, limited to small and shallow contamination areas	Li et al., 2019
Chemical remediation	Thermal remediation	Simple process and thorough treatment	Large energy consumption and secondary pollution	Gong et al., 2018
	Vitrification technique	High efficiency	High cost, limited to small soil area, treated land and soil losing environmental functions	Dhaliwal et al., 2020
	Chemical fixation	Fast to implement, high efficiency	High cost and limited application site	Nejad et al., 2018
	Electrokinetic remediation	Economical and efficient	Limited to low permeability soils	Singh and Prasad, 2015
Bioremediation	Phytoremediation	Low cost, eco-friendly, almost no side effects	Slow process, low efficiency, and long cycle	Liu et al., 2020
Microbial bioremediation	Remove the contaminants, soils retain their properties and could be replaced on the reclaimed site	Microbes are easily affected by soil’s physical and chemical properties	Grover et al., 2021

The current review has discussed the underlying mechanisms of PGPB involved in the heavy metal bioremediation in response to metal alone or in combination with climatic conditions (e.g., drought, salt, and heat) have been discussed. The main objective is to provide an overview of recent advances in developing PGPB-assisted phytoremediation under various climatic stresses, including the strategies to improve remediating plants tolerance and biomass, metal detoxification, bioaccumulation, transformation, and translocation activities. This review also emphasizes the commercial application of PGPB to improve phytoremediation efficiency.

### Metal-resistant plant growth-promoting bacteria

**Figure 1:**
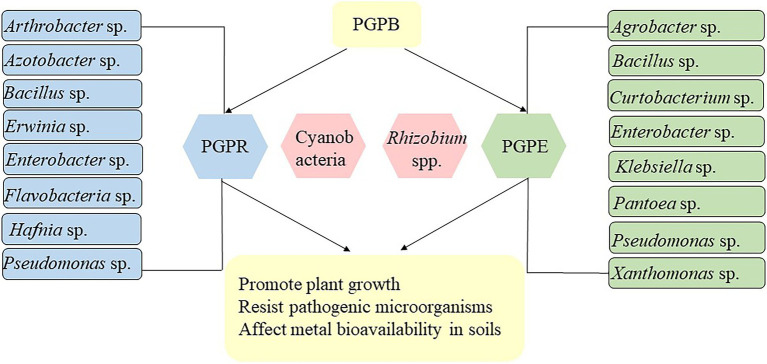
Type of plant growth-promoting bacteria (Glick et al., 2012).

Plant growth-promoting bacteria are a type of bacteria that may colonize rhizosphere soils and plant tissues and stimulate plant growth through various plant growth-promoting (PGP) activities under different conditions (Hashem et al., 2019). PGPB can improve plant abiotic and biotic stress tolerance by directly modulating phytohormone levels and facilitating resource acquisition, and/or indirectly by protecting plants against phytopathogens through the production of antibiotics and siderophores (Backer et al., 2018). They may act as free-living or rhizosphere bacteria (that form specific symbiotic relationships with roots), endophytic bacteria (that can colonize plant interior tissues), *Rhizobia* spp., and cyanobacteria (Glick et al., 2012). Figure 1 shows the type of plant growth-promoting bacteria. They all use the same PGP methods (direct and indirect); however, there are distinctions among these bacteria. Endophytic bacteria are more valuable in real-world applications than rhizobacteria because of their stable living environment and closer contact with plants for nutrient supply (Afzal et al., 2019). It has been reported that a group of metal-resistant PGPB, such as *Pseudomonas*, *Arthrobacter*, *Agrobacterium*, *Bacillus*, *Azoarcus*, *Azospirillum*, *Azotobacter*, *Burkholderia*, *Klebsiella*, *Alcaligenes*, *Serratia*, *Rhizobium,* and *Enterobacter* species have great potential to promote the growth of various plants in the metal-contaminated environments (Enebe and Babalola, 2018). These metal-resistant PGPB were found to enhance plant metal tolerance by improving detoxification rates of plants, enzymes secreted by plant roots, and soil pH modification (Guo et al., 2020). Moreover, certain metal-resistant PGPB can also alter metal mobility and bioavailability, and consequently plant usage rate by releasing chelating agents, acidification, and redox changes (Verma and Kuila, 2019). Therefore, these metal-resistant PGPB strains can be used as a suitable candidate for metal phytoremediation to minimize the adverse impact of metals and enhance metal accumulation capacity of plants. A number of metal-resistant PGPB have been reported to improve plant bioaccumulation/phytoextraction capacity through the secretion of siderophores and organic acids, which improve metal bioavailability by reducing soil pH (Manoj et al., 2020). In contrast, some metal-resistant PGPB can release polymeric substances (such as glomalin and polysaccharides) that speed up metal phytostabilization by limiting their mobility (Ma et al., 2016a).

However, microbes have a strong dependence on the environment, and changes in environmental conditions can modulate the diversity, abundance, and functioning of bacteria (Afzal et al., 2019). Sánchez-Marañón et al. (2017) studied bacterial communities in eight soils selected along a soil-forming gradient and found that distinct bacterial distributions were positively connected to organic carbon, water-stable aggregates, porosity, water, and acidity. Furthermore, endophytic microbiota can be influenced by the age, genotype, nutritional status, and geographical location of the host plants (Ahmed et al., 2020). Carvalhais et al. (2013) confirmed that the transcriptional changes of *B. amyloliquefaciens* caused by the nutrient-deficient corn exudates were significantly correlated with the concentrations of amino acids, aspartic acid, valine, and glutamic acid in the root exudates. Furthermore, differences in microbial communities can result from host plant preferences for stress conditions. Wu et al. (2020a) reported the growth of *B. subtilis* at high salinity reduces the cell expansion pressure due to the passage of water through an osmotic gradient. Similarly, Xu et al. (2018) also demonstrated that drought delayed the development of early root microbiota in *Sorghum bicolor.* Nevertheless, inoculation of climatic stress-resistant PGPB has great potential to increase plant resistance/tolerance to such environmental stresses. Bruno et al. (2020) pointed out that *Bacillus cereus* improved the growth of *S. bicolor* and its phytoremediation potential in Cr-contaminated soil at elevated atmospheric temperature by producing siderophores and indole-3-acetic acid (IAA).

## Mechanisms of PGPB in remediation of metal-contaminated soils under various climatic stresses

**Figure 2:**
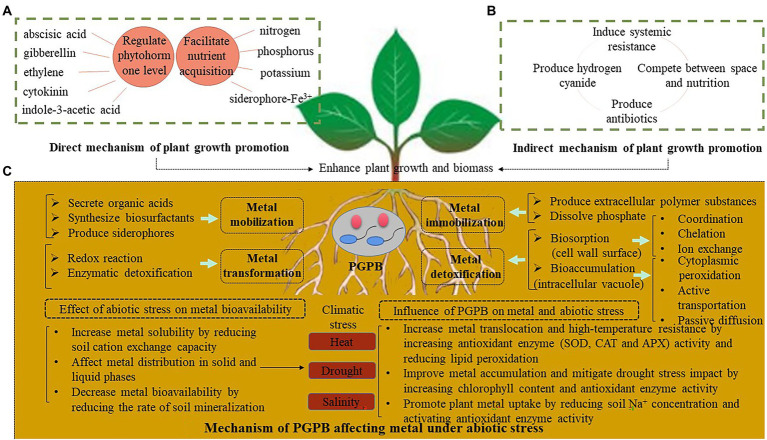
Mechanism of the synergistic effects of PGPB on the bioremediation of metal-contaminated soils. **(A)** Direct mechanism of plant growth promotion; **(B)** Indirect mechanism of plant growth promotion; **(C)** Mechanism of PGPB affecting metal under abiotic stress.

The microbiome is essential for plant growth and function, particularly PGPB play a key role in plant growth regulation *via* phytohormone production, plant nutrient acquisition, and abiotic and biotic stress alleviation, which enable plants to tolerate high concentrations of heavy metals and thus better survive in challenging conditions. Figure 2 depicts the mechanism of the synergistic effect of PGPB on the phytoremediation of metal-contaminated soils. PGPB could promote plant growth directly and/or indirectly under metal stress. The direct plant growth promotion by PGPB involves producing phytohormones (e.g., auxin, cytokinin, gibberellin, abscisic acid, and ethylene), or facilitating plant nutrient uptake (e.g., nitrogen, phosphorus, potassium, etc.; Figure 2A). Since the interaction between the antibacterial activity of PGPB and nutrient competition inhibits the growth of pathogenic bacteria, the production of antibacterial compounds and the coexistence of pathogens enhance ISR and indirectly promote plant growth (Figure 2B). One or more of these mechanisms can be used by specific PGPB to enhance plant resistance to environmental stresses. PGPB also can adsorb metals through coordinate, chelate, and ion exchange. Under climatic stresses, PGPB can also effectively change metal bioavailability through mobilization, stabilization, and transformation, thereby improving bioremediation efficiency and reducing the climatic stress effect by regulating the antioxidant enzyme activity and ion balance in plants (Figure 2C).

### Resistance mechanism of PGPB in alleviating stress in plants

The biomass of remediating plants and soil metal bioavailability are key factors influencing phytoremediation efficiency. Nitrogen, phosphorus, potassium, and other minerals are essential nutrients for plant growth. Although there are many phosphorus and potassium elements present in soils, most of them are insoluble and not bioavailable for plants. The existence of metals can aggravate the loss of nutrients in soils, making them unable to be effectively absorbed and utilized by plants (Ashraf et al., 2017; Ahemad, 2019). Metals can also have a significant impact on plant growth and development. Many studies have shown that when plants are grown under metal stress conditions, the membrane system of plants is damaged, and then the structure and function of organelles are affected, and various physiological and biochemical processes (such as chlorophyll content, photosynthesis rate, biomass reduction, and so on) in their tissues are impaired (Zhu et al., 2020). In metal-contaminated soil, PGPB can improve plant tolerance to such stresses (metal and other climatic stresses) and stimulate plant growth by maintaining nutrient status and adjusting phytohormonal balance through the production of plant growth regulators. Several studies have indicated that PGPB contributes significantly to phytohormone production, which can not only regulate plant growth, regular development, and physiological processes and but also control biological and non-biological stress responses (Afzal et al., 2019; Hewage et al., 2020). Chen et al. (2017) reported that under the stress of Zn and Cd, *Pseudomonas fluorescens* can promote the growth and physiological indicators (above ground chlorophyll and enzyme activity) of *Sedum alfredii* by producing IAA, and improve plant Cd absorption by regulating the expression and transport genes of Cd. Furthermore, some IAA-producing PGPB could increase plant uptake of nutrients and water and reduce the stress effects of salt and drought on plants by changing the roots system architecture (Etesami and Maheshwari, 2018). Pan et al. (2019) demonstrated the potential of abscisic acid (ABA)-producing *B. subtilis* to minimize Cd accumulation in *Arabidopsis thaliana*. A number of bacteria with gibberellin acid production capacity alleviate metal toxicity by reducing Cd uptake and lipid peroxidation, altering hormonal balance, and regulating activities of proteases, catalase, and peroxidase (Etesami, 2018). Moreover, PGPB containing ACCD can help plants to cleave the synthesis ethylene precursor 1-aminocyclopropane-1-carboxylate by metabolizing it into α-butanone acid and ammonia, thereby alleviating the ethylene level in plants and improving their climatic stress tolerance (Vejan et al., 2016).

Microorganisms can also improve plant growth directly *via* nitrogen fixation, phosphorus dissolution, and potassium dissolution (Ashraf et al., 2017). *Stenotrophomonas rhizophila* and *B. amyloliquefaciens* were able to fix nitrogen, thus providing abundant nitrogen to *Brassica napus* (Liu et al., 2021). Besides, certain PGPB like *Klebsiella variicola* can convert insoluble phosphate to soluble forms through the secretion of enzymes (phosphonates and C–P lyases) and organic acids (citric acid oxalic, fumaric, and malic; Manoj et al., 2020), thereby improving the phosphorus availability in the rhizosphere under metal stress. Potassium-solubilizing bacteria can form biofilms on the surface of rhizosphere minerals by producing capsular polysaccharides, hydroxyl anions, iron carriers, and extracellular enzymes, as well as dissolve K-containing minerals in soils and effectively release K by synthesizing organic and inorganic acids (Etesami and Maheshwari, 2018). Many recent investigations have revealed that a number of PGPB, namely *Pseudomonas*, *Bacillus*, *Klebsiella*, and *Pantoea*, can release K from insoluble minerals such as mica and illite through a variety of mechanisms (including acidolysis, chelation, exchange reactions, and complexolysis), including the production of organic acids (Bakhshandeh et al., 2017). Some microorganisms can absorb iron from the siderophore-Fe complex through chelation degradation and release of iron, direct uptake of siderophore-Fe complex and ligand exchange (Ma et al., 2016b). Agarwal et al. (2020) found that *Staphylococcus warneri* GL1, *B. velezensis* GL3, GL5, and GMC2, isolated from *Gnetum gnemon*, are able to secrete various siderophores with high affinity for Fe^3+^, effectively inhibiting the growth of *Ralstonia solanacearum* in the rhizosphere.

Over the past 2 decades, there has been a better understanding of antibiotics as the basis of the biological control mechanism of PGPB and a variety of antibiotics have been identified such as amphibious steroids, 2,4-diacetylphloroglucinol, hydrogen cyanide (HCN), oomycin A, phenazine, pyoluteorin, pyrrolidin tensin, and troponin (Compant et al., 2005). *Pseudomonas fluorescens* can inhibit the root rot of *Nicotiana tabacum* caused by *Thielaviopsis basicola* by synthesizing pyocyanin and 2,4-diacetyl fluorescein (Morales-Cedeño et al., 2021). Competition is one of the important mechanisms of PGPB’s resistance, including nutrition competition and locus competition. Through high-density colonization in plant rhizosphere or tissues, PGPB competes with indigenous microorganisms in the same micro-environment for oxygen, nutrition, and space (Compant et al., 2005). Induced systemic resistance (ISR) is the term being used for microbe-mediated induce plant resistance to infection by pathogenic fungi, bacteria, viruses, nematodes, and pests (Manoj et al., 2020). ISR is a central mechanism for *Pseudomonas*, *Trichoderma*, and *Bacillus* to protect plant against various pathogens (Saeed et al., 2021). In addition, lipopolysaccharide, flagellum and siderophores produced by PGPB can also cause ISR in plants (Ahmed et al., 2020). Inoculation with hydrogen cyanide-producing *Brevibacterium casei* MH8a significantly increased the biomass and accumulation of Cd, Zn, and Cu in *Sinapis alba* (Plociniczak et al., 2016). This is probably due to the potential of bacterial HCN to enhance plant growth and metal mobilization (Manoj et al., 2020). Recently, the release of stress-related volatile compounds was also found to increase plant biomass, yield, and survival under water stress (Etesami and Glick, 2020).

### Mechanism of action of PGPB on heavy metals

Climatic stresses have a significant impact on metal bioavailability. For instance, extreme temperatures can disrupt the nutrient and metal pathway by dissolving organic matter, decomposing microbial cells and destroying soil aggregates, and altering metal bioavailability, absorption, and distribution in plant tissues (Rajkumar et al., 2013). Li et al. (2011) found that higher temperature increased Cd accumulation in roots while decreased root elongation of *Triticum aestivum* in Cd-contaminated soils. This is because higher temperatures increased Cd toxicity to plant roots by increasing Cd accumulation and changing the subcellular distribution of Cd. Furthermore, changes in soil moisture can affect soil pH, EH, calcium carbonate, soluble organic content, and the electrochemical characteristics of the soil surface, all of which have an indirect impact on metal distribution in the soil solid–liquid phase and thus metal bioavailability. Pascual et al. (2004) noticed lower bioavailability of Zn, Cu, Mn, and Ni concentrations in soils when water was scarce. This was ascribed to a lower rate of soil mineralization as a result of drought stress. In response, increased metal deposition in the leaves has the ability to improve drought stress resistance and delay the negative consequences by reducing stratum corneum transpiration or increasing osmotic pressure in cells (Rajkumar et al., 2013).

#### Metal mobilization

Heavy metals are commonly present in both bioavailable and non-bioavailable forms in soils. The mobility and solubility of metals in soils are considered to be important factors influencing plant extraction efficiency (Ma et al., 2016b). Metal-resistant PGPB can mobilize metals and thus increase metal availability in the soil environment by secreting various organic acids (such as oxalic acid, acetic acid, and citric acid) and biosurfactants. Wu et al. (2018) noted that the inoculation of *Buttiauxella* sp. SaSR13 increased the content of root exudates (especially malic and oxalic acids) of *S. alfredii*, resulting in significant increases in the bioavailability and plant uptake of Cd. Additionally, biosurfactants can promote the entry of hydrophobic contaminants into the aqueous phase by solubilizing and micellizing the contaminants. These micelles and dissolved contaminants allow metal removal *via* soil washing or make them easily absorbed by plants (Akbari et al., 2018). Therefore, biosurfactant-producing microorganisms present in contaminated soils can effectively enhance metal mobility (Lal et al., 2018). San Martín et al. (2021) demonstrated that rhamnolipid-producing *Pseudomonas* Y3-B1A achieved a maximum vanadium removal efficiency of 85.5%.

Metal-resistant PGPB can also mobilize metal through biomethylation, leading to their volatilization. Certain PGPB can transfer methyl groups to metals (Pb and Se, etc.) to form methylated metal compounds with altered volatility, solubility, and toxicity (Ahemad, 2019). Microbial methylation of arsenic (As) raises trace levels of As species like monomethylarsinic acid and dimethylarsinic acid (DMA) in soils. Methylated As is absorbed by plant roots more slowly than inorganic As. DMA does not form compounds with plant chelating agents and is not quenched in vacuoles, allowing for efficient DMA transport inside the plants (Bali and Sidhu, 2021). Zhang et al. (2015) also proposed that As trioxide S-adenosylmethionine methyltransferase of *Pseudomonas alcaligenes* play a major role in the methylation and detoxification of As (III), which can be used in the bioremediation of As-contaminated environment.

#### Metal stabilization

The combination of metals with extracellular substances (e.g., anionic functional groups and extracellular polymers) can reduce metal bioavailability in soils, therefore reducing metal absorption or migration to aboveground plant parts. For instance, many metals can be efficiently immobilized in soils by combining with anionic functional groups on the cell surface (e.g., mercapto, carboxyl, hydroxyl, sulfonate, amine, and amide groups; Jacob et al., 2018). These substances reduce metal toxicity by forming complexes or effective barriers around cells (Ahemad, 2019). Some PGPB can also reduce metal bioavailability in soils through precipitation, alkalization, and complexation processes. The inorganic acids secreted by PGPB (e.g., hydrogen sulfide, bicarbonate, and phosphate) can also react rapidly with certain dissolved metals (Cu, Fe, Zn, and Pb) to form insoluble precipitates (Ma et al., 2016b).

Extracellular polymer substances (EPS) produced by PGPB are biosynthetic polymers composed mainly of polysaccharides, proteins, uronic acid, hummus, lipids, and other compounds. A previous study noted that bacterial EPS play a variety of biological activities in microbes and plants (Manoj et al., 2020). EPS generated by PGPB was shown to bind firmly to potentially harmful trace elements and capture precipitated metal sulfides and oxides to form organic metal complexes, enhancing resistance to toxic trace elements (Etesami and Maheshwari, 2018). The role of PGPB in the biosorption of Cs^+^ was confirmed by the production of EPS and biofilm formation of *Nocardiopsis* sp. (Sivaperumal et al., 2018). Silambarasan et al. (2019b) discovered that *Rhodotorula* sp. CAH2 could survive up to 6 mmol L^−1^ Al and produce EPS consisting of glucose, mannose, and galactose even under multiple stress conditions (salt and drought), along with the yield increasing as the stress level increased.

Phosphate solubilized by PGPB can also precipitate metals as metal accumulation in bacterial biomass is mediated by phosphatases that release inorganic phosphates from supplied organophosphate donor molecules (e.g., glycerol 2-phosphate) and metal cations precipitate on the biomass as phosphates (Gadd, 2004). In addition, microbial-induced carbonate precipitation (MICP) has been proposed as a viable bioremediation approach for metal immobilization. In MICP, carbonates can bind to the metals (e.g., Pb^2+^ and Cu^2+^) on the surface, after which these metal elements change from soluble forms to insoluble forms, thus reducing their toxicity (Tamayo-Figueroa et al., 2019). The MICP caused by *B. pasteurii* ATCC 11859 maintained the microbial growth while reducing the available Pb content in the soil, resulting in a decrease in Pb extraction and available Pb content by 76.34 and 41.65%, respectively (Chen et al., 2021).

#### Metal transformation

The valence state of the metal determines its toxicity. The oxidation–reduction process of metal by bacteria results in various chemical transformations of metal, affecting their shape and mobility in soils, which is regarded as an essential detoxifying mechanism (Ma et al., 2016b). Metal ions’ redox reactions can be regulated by PGPB *via* cell metabolism, and metals could be converted into non-bioavailable states in the rhizosphere to reduce their toxic effects on plants (Sharma, 2021). It has been found that variation in metal-reducing bacteria can catalyze the reduction reaction and use metal to replace Fe^3+^ and S^0^ as terminal electron receptors in anaerobic respiration (Yin et al., 2019).

Enzyme-mediated reduction in toxic metals to less destructive forms is another popular strategy for reducing metal toxicity, which helps to improve microbial resistance to metal ions. These enzymes cleave bonds and use the energy generated by biochemical reactions to assist in the transfer of electrons from one compound to another (Voica et al., 2016). Harmful pollutants are eventually oxidized to innocuous molecules as a result of these processes. Furthermore, these enzymes aid in the humification of various phenolic compounds produced by lignin degradation in the soil environment, as well as the detoxification of various xenobiotics, such as aniline or phenolic compounds *via* chemical interactions (Jacob et al., 2018). Giovanella et al. (2016) found that *Pseudomonas* B50A removed 86% of Hg in the medium. This is probably due to the fact that Hg (II) reductase produced by strain B50A could effectively reduce Hg (II) to Hg (0) to reduce its toxicity.

#### Metal detoxification

The metal detoxification processes induced by PGPB have a significant influence on phytoremediation efficiency. Metal biosorption and bioaccumulation by bacteria, as well as the synthesis of plant hormones, ACCD, and other secretions, have all been proven to improve plant resistance to metals (Etesami and Maheshwari, 2018; Yaashikaa et al., 2021). Biosorption and bioaccumulation of inorganic and organic pollutants are determined by interaction traits of biomass and concentration of pollutants. In biosorption, the contaminants adhere to the surface of the cell wall, whereas in bioaccumulation, the contaminants accumulate within the cells (Ma et al., 2016b; Priyadarshanee and Das, 2021). Huang et al. (2020a) found that *B. subtilis* had significant biosorption potential, since it adsorbed about 10–20 mg L^−1^ concentration of Cd^2+^. This type of biosorption phenomenon can reduce the pollutants (especially metals) toxicity to plants. In another study, Sedlakova-Kadukova et al. (2019) proved that *Streptomyces* K11 isolated from alkaline brown mud disposal site considerably reduced the Zn toxicity through extracellular accumulation and chelation, which are related to its Zn tolerance and high bioaccumulation efficiency.

## Genetically modified organisms for bioremediation

The use of genetically engineered microorganisms (GEMs) is also a promising strategy to clean up metal-polluted soils. Transgenic methods are not only used to convert functional genes but also to elevate particularly recognized promoters to existing gene functions connected with metal accumulation/translocation/detoxification mechanisms and introduce them to target bacteria (Pratush et al., 2018). As a low molecular weight, cysteine-rich protein, metallothioneins (MTs) found in many bacteria have the ability to bind metals and form complex biochemical structures (Venegas-Rioseco et al., 2022). Some gene transformation experiments have convincingly demonstrated that MT_S_ produced by PGPB (e.g., *P. putida* and *Mycobacterium tuberculosis*) can improve plant tolerance to metals (Mierek-Adamska et al., 2017; Nanda et al., 2019). Li et al. (2021) proved that *E. coli* cells expressing SUMO-*Sh*MT3 bioaccumulated Cd^2+^, Cu^2+^, and Zn^2+^. The biofilm-forming marine bacterium *P. aeruginosa* N6P6 possessing the *bmtA* gene resisted a variety of metals (e.g., Pb, Cd, Hg, Cr, and Zn; Kumari and Das, 2019).

Since several elements in the promoter region of the metals responsive gene can be activated by plant hormones and growth regulators, the relationship between these regulator compounds and metal chelator phytochelatins is very important, which are in the first line of heavy metal defense mechanism is critical (Pal et al., 2018). Phytochelatins are enzymatically synthesized from glutathione by phytochelatin synthase activity in the presence of metal and their synthesis also initiates/transforms the entities of metal anions (Ag, Au, Cd, Cu, Hg, Pb, and Zn) and cations (As; Ozyigit et al., 2020). de Souza and Vicente (2020) proved that recombinant *E. coli* clones expressing the synthetic phytochelatin EC20 have higher Cd^2+^ biosorption capacity and tolerance than that without EC20. Heavy-metal ATPases (HMA), a subfamily of P-type HMA transporters, are found in a wide range of microorganisms. The energy released by ATP hydrolysis is mostly used to power the transmembrane transport of some metal ions, such as Ag^+^, Zn^2+^, Cd^2+^, Cu^2+^, and Ni^2+^ (Yang et al., 2022). Begum et al. (2019) showed that *HSP70* and *HMA3* genes (a member of the HMA family) were highly expressed in *Panicum virgatum* inoculated with *P grimontii* and *P. vagans* under Cd stress, resulting in an increase in the biomass and IAA yield in inoculated plants, but a decrease in Cd accumulation. Both the natural resistance-associated macrophage protein family and the yellow streak-like transporters are also responsible for the absorption, transport, and detoxification of transition metals (Chowdhury et al., 2021; Yang et al., 2022).

The application of these GEMs is a very effective method to remove pollutants from the environment. However, the application of GEMs can affect the natural ecosystem, posing risks to the environment (Hussain et al., 2018). GEMs are considered a competitive alien species to the ecosystem and their introduction may reduce microbial biodiversity in the ecosystem. Besides, they may have adverse effects on human health, causing cancer and other genetic diseases (Saravanan et al., 2022). Undoubtedly, GEMs have potential ecological risks, but it is possible to find an efficient way to implement GEMs in the bioremediation of metal-polluted soils in the future through technical safeguards and innovation. For instance, some countries have issued necessary guidelines for assessing and monitoring the risks of GEMs in the environment, emphasizing risk stratification when applying GEMs for bioremediation (Wu et al., 2021). There have been recent attempts to design and track GEMs including the development of a set of criteria for the utilization of GEMs (Ezezika and Singer, 2010). In addition, another containment approach mainly involves designing “suicidal GEMs,” when the pollutants are degraded, the killer gene is activated and the GEMs are then eradicated (Rebello et al., 2021).

## Application and commercialization of PGPB in phytoremediation under environmental stress

The use of PGPB in bioremediation has become more and more popular due to their abilities to detoxify and degrade toxins and promote plant growth. Silambarasan et al. (2020) demonstrated that the inoculation with *Pseudomonas citronellolis* SLP6 improved bud and root growth (length, fresh, and dried biomass), chlorophyll content, antioxidant enzyme activity, and Cu uptake in roots and shoots under Cu and Cu+ salt stress. Interestingly, they concluded that the *P. citronellolis* SLP6 amalgamation could be an effective approach for phytostabilization in Cu-contaminated saline soils. Accordingly, Bruno et al. (2021) reported that the inoculation of multi-metal (MM) and increased atmospheric temperature (IAT) tolerant *B. cereus* TCU11 significantly improved the growth and phytoextraction (Pb, Zn, Ni, Cu, and Cd) potential of *Zea mays* in metal-contaminated soils. However, most PGPB applications in metal bioremediation are done in pot or greenhouse experiments, with *in situ* investigations in field conditions being rare. The field experiment conducted by Ren et al. (2019) showed that *B. cepacia* J62 increased the contents of ascorbic acid and glutathione in *B. napus* and reduced the oxidative stress caused by metals. The successful colonization contributed to increasing the biomass and the total Cu absorption (67.91%). Prapagdee and Khonsue (2015) found that the Cd accumulation in the root, ground tissue, and whole plant increased by 1.2-, 1.4-, and 1.1-fold, respectively, after inoculation of *Arthrobacter* sp. with *Ocimum gratissimum* for 2 months in the field conditions. The applications of PGPB in metal phytoremediation in the past 5 years are summarized in Table 3.

**Table 3:** Application of PGPB in bioremediation under environmental stress.

PGPB strain	Metal	Abiotic stress	Host plant	PGP trait	Remarks	Phytoremediation method	Experimental condition	Reference
*Pseudomonas fluorescens*, *Luteibacter* sp., and *Variovorax* sp.	Pb	-	*Lathyrus sativus*	IAA, siderophores	Improved the photosynthetic pigments biosynthesis, membrane stability, and the accumulation of proline and soluble sugars; Increased Pb tolerance and accumulation in plants	Phytoextraction	Pot experiment	Abdelkrim et al., 2018
*Bacillus* sp. CIK-516	Ni	-	*Raphanus sativus*	IAA, ACCD, and EPS	Increased plant biomass, chlorophyll and nitrogen contents, and Ni uptake	Phytoextraction	Pot experiment	Akhtar et al., 2018
*Streptomyces pactum* Act 12	Cd, Cu, Zn, and Pb	-	*Triticum aestivum*	IAA, siderophores, ACCD	Increased plants biomass and the uptake of Cd, Cu, and Zn in shoots and roots; Decreased antioxidant activities and lipid peroxidation	Phytoextraction	Pot experiment	Ali et al., 2021
*Bacillus* sp. SB1*, Halobacillus* sp. SB2	Zn, Al, Pb	Salinity	*Arachis hypogaea*	N fixation, P solubilization	Promoted plant growth and reduced Zn, Al, Pb toxicity to the seedlings	Phytostabilization	Petri dish experiment	Banik et al., 2018
*B. cereus* TCU11	Pb, Zn, Ni, Cu, Cd	High temperature	*Zea mays*	IAA, siderophores	Increased plant biomass, chlorophyll, carotenoid and protein contents, and Pb, Zn, Ni, Cu, and Cd accumulations in plant tissues, and their translocation from root to bud	Phytoextraction	Pot experiment	Bruno et al., 2021
*B. cereus* TCR17*, Providencia rettgeri* TCR21, *Myroides odoratimimus* TCR22	Cr	High temperature	*Sorghum bicolor*	IAA, siderophores	Increased the crown length, root length, plant fresh and dry weight, and antioxidant status (SOD, CAT, and APX); Reduced proline, MDA content, and Cr accumulation in plants	Phytostabilization	Pot experiment	Bruno et al., 2020
*Variovorax* sp., *Micrococcus* sp., *Microbacterium* sp.	Zn, Cd	-	*Noccaea caerulescens*, *Rumex acetosa*	IAA, ACCD, P solubilization, siderophores	Increased chlorophyll, carotenoid contents, and soil nutrient cycling; Facilitated Zn and Cd translocation in plants	Phytostabilization	Pot experiment	Burges et al., 2017
*Sphingomonas* sp. C40	Cd	-	*Oryza sativa*	IAA, siderophores	Successfully colonized the rhizosphere soils and root interiors; Increased plant biomass and root polyamine production and their related gene expression; and Reduced Cd accumulation and translocation from roots to grains	Phytostabilization	Pot experiment	Cheng et al., 2021
*B. aryabhattai* AS6	As	-	*O. Sativa*	N fixation, IAA, P solubilization, siderophores, ACCD, and EPS	Improved plant biomass and SOD and CAT activities; Ameliorated As toxicity in plants; and Exhibited bio-removal and bioaccumulation of As	Phytoextraction	Pot experiment	Ghosh et al., 2018
*Bacillus* sp. QX8 and QX13	Cd, Pb	-	*Solanum nigrum*	IAA, siderophores, ACCD, P solubilization	Increased plant biomass, enzymatic activity, and Cd and Pb accumulation by plants	Phytoextraction	Pot experiment	He et al., 2020
*B. cereus* HM5, *B. thuringiensis* HM7	Mn	-	*Broussonetia papyrifera*	IAA, P solubilization, siderophores	Increased plant biomass, total root length, surface area, and Mn bioavailability in soils; Inhibited plant lipid peroxidation; Decreased MDA content, antioxidant enzyme activity in leaves, and the toxic effect of Mn on plants	Phytoextraction	Pot experiment	Huang et al., 2020b
*Serratia* sp. ZTB	Zn	-	*Z. mays*	IAA, ACCD, siderophores, and P and K solubilization	Decreased Zn phytotoxicity; Improved plant growth and Zn accumulation in host plants	Phytostabilization	Pot experiment	Jain et al., 2020
*S. pactum* Act12, *B. subtilis, B. licheniformis*	Cd, Zn	-	*Brassica juncea*	P solubilization	Promoted microbial community, enzymes activity, plant biomasses, and accumulation of Cd and Zn	Phytoextraction	Pot experiment	Jeyasundar et al., 2021
*Acinetobacter* sp. RA1, *Bacillus* sp. EhS7*, Bacillus* sp. RA2	Cu, Cd	-	*Perennial ryegrass*	IAA, siderophores, P solubilization	Increased the shoot and root biomass; Reduced SOD activity, MDA content, and Cu, Cd transfer to the above-ground parts	Phytostabilization	Pot experiment	Ke et al., 2021
*P. azotoformans* ASS1	Cu, Zn, Ni	Drought	*Trifolium arvense*	ACCD, siderophores, N fixation, P solubilization	Increased chlorophyll content of plants, accumulation of antioxidant enzymes (CAT, POD and SOD), and Cu, Zn, Ni uptake of *T. arvense*; Reduced proline accumulation and oxidative damage of membrane lipids of host plants	Phytostabilization	Pot experiment	Ma et al., 2017
*Bacillus* sp. TZ5	Cd	-	*Lolium perenne*	IAA, P solubilization	Colonized well in soils and increased plant biomass; Decreased Cd accumulation in ryegrass	Phytostabilization	Pot experiment	Ma et al., 2020
*B. atrophaeus* GQJK17 S8, *E. asburiae* QB1	Cu, Cd	Salinity	*Chenopodium quinoa willd.*	IAA, siderophores, P solubilization	Improved the germination rate, seedling biomass and growth vigor index, and plant tolerance to Cu and Cd	-	Petri dish experiment	Mahdi et al., 2021
*Kocuria flava* AB402, *B. vietnamensis* AB403	Cu, Cr, Ni, Zn, Co, Cd	-	*O. sativa*	IAA, siderophores, EPS	Colonized successfully in rice plant root; Enhanced plant growth; Decreased As uptake and accumulation in plants	Phytostabilization	Pot experiment	Mallick et al., 2018
*A. baumannii* BacI43*, Pseudomonas* sp. BacI7	Hg	-	*Z. mays*	Siderophores, P solubilization	Enhanced total dry biomass; Increased total Hg bioaccumulation and volatilization; Reduced soil Hg content	Phytovolatilization	Pot experiment	Mello et al., 2020
*B. safensis* FO-036b(T), *P. fluorescens* p.f.169 (along with SiO_2_ and zeolite NPs)	Pb, Zn	-	*Helianthus annuus*	IAA, siderophores, ACCD	Promoted plant growth; Reduced the accumulation of Pb and Zn in plant tissues	Phytoextraction	Pot experiment	Mousavi et al., 2018
*Rhodobacter sphaeroides*	Cd, Zn	-	*T. aestivum*	IAA	Enhanced the wheat cellular homeostasis; Reduced the accumulation of Cd and Zn in plants	Phytostabilization	Pot experiment	Peng et al., 2018
*Pseudomonas* sp. K32	Cd	-	*O. Sativa*	IAA, N fixation, P solubilization	Increased total chlorophyll content, amylase activity, total sugar content; Decreased MDA content and Cd uptake	Phytostabilization	Hydroponic cultivation	Pramanik et al., 2021
*Arthrobacter* sp. TISTR 2220	Cd		*Ocimum gratissimum*	IAA	Enhance Cd accumulation and translocation of Cd from plant roots to the shoots during a 2-month harvest period	Phytoextraction	Field trial experiment	Prapagdee and Khonsue, 2015
*Proteus* sp. DSP1*, Pseudomonas* sp. DSP17, *Ensifer meliloti* RhOL6	Cu, Pb, Zn	High temperatures	*Medicago sativa*	IAA, siderophores, N fixation, P solubilization	Colonized plant root system; Enhanced plant growth, synthesized non-enzymatic metabolites and enzymes; and Decreased metal (Cu, Pb, and Zn) translocation to shoots	Phytostabilization	Pot experiment	Raklami et al., 2019
*Microbacterium oxydans* JYC17, *P. thivervalensis* Y1-3-9, and *B. cepacia* J62	Cu		*Brassica napus*	IAA, ACCD, siderophores, P solubilization	Colonized plant rhizosphere and endosphere; Enhanced plant biomass and Cu uptake; Decreased POD activity	Phytoextraction	Field trial experiment	Ren et al., 2019
*P. aeruginosa* CPSB1	Cu, Cr, Cd	-	*T. aestivum*	ACCD, IAA, HCN, siderophore, P solubilization	Enhanced root dry biomass, shoot and spikes; Decreased the levels of proline, antioxidant enzymes, MDA content, and metal (Cu, Cr, and Cd) uptake by plants	Phytostabilization	Pot experiment	Rizvi and Khan, 2017
*Curtobacterium herbarum* CAH5	Al	Drought	*Lactuca sativa*	IAA, ACCD, siderophores, and P solubilization	Enhanced chlorophyll contents and antioxidant enzymes; Reduced MDA content in leaves and Al accumulation in plants; Exhibited bio-removal of Al	Phytostabilization	Pot experiment	Silambarasan et al., 2019c
*Rhodotorula mucilaginosa* CAM4	Al	Drought and salinity	*L. sativa*	IAA, siderophores	Improved plant growth, photosynthetic pigment content and accumulation of antioxidant enzymes; Reduced oxidative stress and Al accumulation in plants	Phytostabilization	Pot experiment	Silambarasan et al., 2019a
*Serratia* sp. CP-13	Cd	-	*Z. mays*	IAA, P solubilization	Increased plant biomass, photosynthetic pigments, antioxidative machinery (SOD, POD, and CAT); Decreased Cd uptake and concomitant lipid peroxidation in plants	Phytostabilization	Petri dish experiment	Tanwir et al., 2021
*Providncia* sp.	Cr	Drought	*Z. mays*	IAA, ACCD, siderophores	Increased plant growth, pigments, protein, phenolics and relative water content; Decreased the lipid peroxidation, proline, superoxide dismutase activity, and Cr translocation	Phytostabilization	Pot experiment	Vishnupradeep et al., 2022
*B. megaterium* H3	Cd, Pb	-	*Brassica rapa, Brassica campestris*	IAA, siderophores	Increased plant biomass, the rhizosphere soil organic matter content and invertase activity; Decreased Cd and Pb translocation factors	Phytostabilization	Pot experiment	Wang et al., 2018
*P. fluorescens*	Cd	-	*Sedum alfredii*	IAA	Increased the formation of lateral roots of its host plants and Cd accumulation in plant roots	Phytoextraction	Pot experiment	Wu et al., 2020b
*B. contaminans* ZCC	Cd	-	*Soy beans*	ACCD, siderophores, organic and inorganic P solubilization, IAA	Promoted plant dry biomass, nitrogen content in above-ground parts, and plant tolerance to Cd; Reduced membrane lipid peroxidation	Phytoextraction	Pot experiment	You et al., 2021
*P. fluorescens* 002	Al	Salinity	*Z. mays*	IAA, ACCD, siderophores	Improved root fresh and dry biomass, chlorophyll, carbohydrate content and the tolerance of plants to Al	-	Petri dish experiment	Zerrouk et al., 2016
*P. plecoglossicida*	Al	Salinity	*Z. mays*	IAA, ACCD	Increased the root length, the number and length of fine roots, the number of lateral roots and the quality of root trunk and the tolerance of plants to Al	-	Pot experiment	Zerrouk et al., 2019

ACCD, 1-aminocyclopropane-1-carboxylate deaminase; APX, ascorbate peroxidase; CAT, catalase; EPS, extracellular polymeric substance; HCN, hydrogen cyanide; IAA, indole-3-acetic acid; MDA, malondialdehyde; N, nitrogen; P, phosphorus; POD, peroxidase; and SOD, superoxide dismutase.

Furthermore, the use of microbial agents in soil bioremediation *via* bioaugmentation techniques would be extremely beneficial to the industrialization of microbial inoculum. Surprisingly, it also boosted the commercialization and market demand for various microbial inoculations. Microbial agents are abundant in high-activity beneficial PGPB, such as N-fixing bacteria and K-dissolving bacteria. These microorganisms’ metabolic activities can effectively reduce metal concentrations and toxicity in the environment (Ma et al., 2016a). Notably, *B. subtilis* has been produced and sold under the trade names RhizoVital® and FZB24® TB for use in alleviating environmental stress and promoting plant growth (Ngalimat et al., 2021). *Pseudomonas fluorescens* has also been used to produce commercial inoculants under the trade names Conquer and Victus (Suyal et al., 2016). Moreover, some inoculant products currently on the market contain several different microorganisms (O’Callaghan et al., 2020). Wang et al. (2020) noted that the combination of *Enterobacter* sp. and *Comamonas* sp. can efficiently fix Cd. In addition, various inorganic materials (mainly clay and talc), organic materials (peat, charcoal, and plant waste materials), and polymers (polysaccharides, protein, and synthetic polymers) were used as carriers for PGPB encapsulation (Szopa et al., 2022). Alginate hydrogels seem to be a successful commercial product. Su et al. (2021) noted that the maximum adsorption capacity of Cu on multilayer calcium alginate beads containing diatom bacteria and *B. Subtilis* can reach 141.34 mg g^−1^. *Bacillus* strains immobilized in alginate macrobeads were also found to enhance drought stress adaptation of *Guinea grass* (Mendoza-Labrador et al., 2021). Tu et al. (2020) used corn stalk biochar and *Pseudomonas* sp. as materials and illustrated the stabilization mechanism of biochar-loaded microbial inoculum on Cd-and Cu-contaminated soils.

## Conclusion and future prospects

This review has addressed the mechanism of PGPB promoting plant growth and enhancing plant resistance under biotic and abiotic stress and the function in response to metal bioavailability and toxicity. This has significant scientific and practical implications for the use of PGPB in the phytoremediation of metal-contaminated soils as well as an understanding of the interaction between external pressure factors and biological processes. However, due to the nature of PGPB themselves, there are limitations in their utilization process. First, the genetic stability of PGPB is poor and easy to change, making them difficult to remove all pollutants. Second, there is a competitive survival relationship between PGPB and indigenous microbes, and eventually, these PGPB strains may be eliminated due to competitive failure. Finally, PGPB are easily affected by other factors such as external environment temperature, soil pH, and so on, thereby hampering the bioremediation efficiency. More research is needed to better understand the interaction between major factors namely metal, soil, microorganisms and plants.

We should pay close attention to how PGPB is employed in actual soil rehabilitation. Soil pH, humidity, and other environmental parameters could affect the efficiency of bioremediation. Compare with the studies of PGPB strain under laboratory conditions, the research on the field and *in situ* remediation experiments of PGPB strain under different environmental conditions is still very scarce.

The effects of different inoculation or application methods of PGPB on the phytoremediation efficiency of metal-polluted soil were explored. The appropriate inoculation method not only can change the soil nutritional status and directly affect PGPB survival and colonization efficiency but also can indirectly affect metal bioavailability in soils by changing the quantity and composition of root exudates of host plants.

There is a need to strengthen the utilization and safe disposal of post-remediation materials. Some bioaccumulating plants could produce certain harmful biomass after soil remediation. If these dry materials cannot be effectively treated, the original significance of bioremediation will be compromised. Therefore, the follow-up treatment of bioremediation technology and the recovery and treatment of metal in soils also have great research value.

It is necessary to develop additional research to analyze and anticipate how metals may influence plant development, metal accumulation, and ecophysiological responses in soils as a result of global climate change. The exact repercussions of climate change on plant–metal interactions in the future are difficult to anticipate due to the complicated interactions between various metals. Furthermore, much of the research lacks information on the behavioral dynamics and metabolomics of PGPB under environmental stresses. As a result, we need to improve our understanding of rhizosphere micro-ecological processes at the molecular level and choose the best couple of plants and PGPB to provide theoretical direction for long-term pollution decontamination.

## Author contributions

YM developed the ideas and wrote the manuscript, and was the project sponsor. YW wrote the first draft of the manuscript. YM, MN, XS, XC, ZL, and DN revised the manuscript. All authors contributed to the article and approved the submitted version.

## Funding

This work is carried out at the College of Resources and Environment, Southwest University, supported by the Fundamental Research Funds for the Central Universities (No. SWU 020010), the Natural Science Foundation of Chongqing (No. cstc2021jcyj-msxmX0827), and Chongqing Returned Overseas Students’ Entrepreneurship and Innovation Support Program (No. cx2021001).

## Conflict of interest

The authors declare that the research was conducted in the absence of any commercial or financial relationships that could be construed as a potential conflict of interest.

## Publisher’s note

All claims expressed in this article are solely those of the authors and do not necessarily represent those of their affiliated organizations, or those of the publisher, the editors and the reviewers. Any product that may be evaluated in this article, or claim that may be made by its manufacturer, is not guaranteed or endorsed by the publisher.
